# Facial Nerve Decompression After Temporal Bone Fracture—The Bangalore Protocol

**DOI:** 10.3389/fneur.2019.01067

**Published:** 2019-10-04

**Authors:** Vijayendra Honnurappa, Vinay Kumar Vijayendra, Nilesh Mahajan, Miriam Redleaf

**Affiliations:** ^1^Vijaya ENT Care Centre, Superspeciality Otology Centre, Bangalore, India; ^2^Department of Otolaryngology-Head and Neck Surgery, University of Illinois Hospitals, Chicago, IL, United States

**Keywords:** facial nerve injury, facial nerve trauma, surgical decompression, facial paralysis, geniculate ganglion

## Abstract

**Background:** Our tertiary otology center treats facial weakness and paralysis after motor vehicle crashes. We evaluate these patients with physical exam, audiogram, Schirmer's test, and CT scan. Our protocol for management of the facial weakness provides good results for our patients.

**Methods:** Our protocol begins with oral steroids, and serial evaluations. Indications for decompression and our unique transcanal approach to identify the sites for decompression are described. A retrospective review of the medical record presents our patients treated between 1998 and 2017.

**Results:** One hundred and forty one patients with grade 4 or more weakness underwent decompression. Mean pre-operative and post-operative House-Brackmann (HB) scores were HB5 and HB2, respectively. Fourteen of 104 patients (13%) presenting with HB5 and 6 still had HB5 or HB6 after decompression. Eighty-three of thee 104 patients (80%) achieved HB1 or HB2 at 6 months. Post-operative bone levels were unchanged. Post-operative air levels were improved in cases of perigeniculate fractures (84%).

**Conclusion:** This Bangalore protocol facilitates advantageous improvement in facial function and conductive hearing loss after traumatic facial nerve crush injuries. The surgical technique, albeit challenging, helps identify the fracture lines, facilitates reconstruction of disrupted ossicles, and avoids craniotomy.

## Introduction

Facial nerve injury after temporal bone fracture usually involves the perigeniculate ganglion area (1, 2). Additional sites may be involved, leading some surgeons to suggest middle cranial fossa, transmastoid, or combined approaches to address those lesions (1–5). It has been noted, however, that the exact site(s) of injury can be difficult to delineate (1).

In Bangalore India, our Center commonly treats injuries after traffic collisions. Our evaluation protocol and surgical approach have served our patients well, in the absence of the access to electrodiagnostic testing. Our decision tree selects whom to explore, and our transcanal approach to the facial nerve exposes the labyrinthine portion of the nerve through the first genu and geniculate ganglion, tympanic segment, second genu, and mastoid segment. This approach enables the surgeon to follow the fracture course directly to the site of lesion, enables addressing the ossicles which are often disrupted in trauma, and avoids craniotomy.

## Methods

IRB approval was obtained.

### Patient Selection

Patients undergo physical examination, House-Brackmann (HB) grading (6), audiometry, CT scan and Schirmer's testing. No electrophysiologic testing is available at this time. Non-surgical treatment is initiated, and the patients' responses indicate who will likely benefit from surgery. In longitudinal fractures, the patients have hematorrhea, tympanic membrane rupture, or hemotympanum with conductive hearing loss. In transverse fractures, the patients have severe giddiness, vomiting, and sensorineural hearing loss. In the majority of cases, the lesion has been found to be around the perigeniculate ganglion area. We find that a positive Schirmer's test (lacrimation absent) in longitudinal fracture indicates probable involvement of the facial nerve proximal to the geniculate ganglion, involving the greater superficial petrosal nerve. If the patient presents with HB4, we proceed to decompression.

If the patient has less than grade 4 at the time of presentation, a trial of oral steroids in a tapering dosage along with physiotherapy is given. These patients are followed for 3 weeks and are then evaluated for improvement of facial function. Patients who achieve eye closure after 3 weeks will achieve complete or near-complete recovery of facial nerve function within 3 months, without surgical intervention. Patients with an initial HB3 who do not achieve eye closure at the end of 3 weeks of conservative management, will undergo surgical intervention. In our experience with this latter scenario, the earlier the intervention, the better will be the final outcome. Best results are achieved if intervention occurs between 3 weeks and 12 weeks. In palsies of 3–6 months', the procedure can be performed with guarded prognosis for improved facial nerve function.

### Surgical Technique

**Figure 1:**
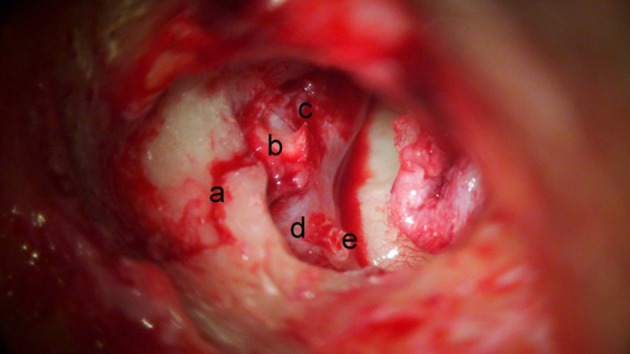
Destructive course of fracture line (right ear). The fracture (a) can be seen extending (b) over the geniculate ganglion, into the perigeniculate ganglion area (c). For orientation horizontal facial nerve (d) and stapes (e) are labeled.

Patients are operated under local anesthesia with sedation or under general anesthesia in the pediatric age group. The mastoid cortex is exposed through a post-auricular incision. Through a transcanal approach, a 270° tympanomeatal flap is raised from 2 through 12 to 6 o'clock in the right ear, and from 10 through 12 to 6 o'clock in the left ear. A wide canalplasty, removing the posterior inferior canal wall, increases exposure while an atticotomy exposes the malleus head and incus body. The fracture lines usually extend from the squamous temporal bone along the posterior canal wall in stepwise fashion toward the middle ear. In most cases, this fracture line dislocates the incudo-malleolar joint and proceeds superior to the supralabyrinthine area—the site most commonly involved in longitudinal fractures. Occasionally, the fracture line can be seen extending over the incudo-stapedial joint, causing its dislocation. Tympanomeatal flap elevation and the boney removal from the canal and attic, allow identification of the destructive route of the fracture line (Figure 1).

**Figure 2:**
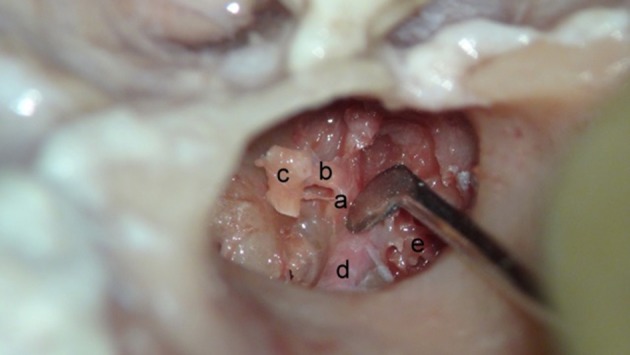
Visualization of the supralabyrinthine region with exposure of the geniculate ganglion (left ear—cadaver). Incus body and malleus head have been removed. The cochleariform process (a) is being down-fractured with the attached tensor tympani tendon (b) and manubrium (c). Horizontal facial nerve (d) and supralabyrinthine area (e) are seen.

**Figure 3:**
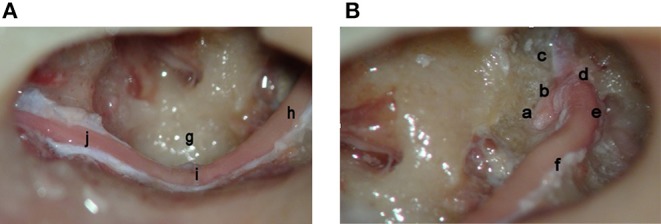
**(A,B)** Complete facial nerve decompression—labyrinthine meatus to stylomastoid foramen (left ear—cadaver). Seen are the meatal foramen (a), labyrinthine portion of the facial nerve (b), greater superficial petrosal nerve (c), the first genu (d), the geniculate ganglion (e), the horizontal segment of the facial nerve (superior image) (f), the sinus tympani (g), the horizontal facial nerve (inferior image) (h), the second genu (i), and the vertical segment out to the stylomastoid foramen (j).

The incudo-stapedial joint is disarticulated, and the incus is removed and preserved (As our results will later show, the majority of these fractures involve the peri-geniculate region which can be reached through the epitympanum, but not via a transmastoid approach. In addition, as our hearing outcomes will later show, the ossicles are already disrupted by the trauma in most of these peri-geniculate cases). The malleus head is amputated and the manubrium is retained with the tympanomeatal flap. The boney removal in the attic is further extended to expose the supralabyrinthine region. The cochleariform process is fractured to expose the geniculate ganglion beneath it (Figure 2). Fragments of the supralabyrinthine cells, which typically impinge into the perigeniculate ganglion area, are removed meticulously with the drill and variously sized curettes, to decompress the labyrinthine segment, first genu and greater superficial petrosal nerve (Figure 3). The route of the labyrinthine facial canal is slowly exposed using a small diamond burr, accessed either medial-anterior-superior or medial-anterior-inferior to the geniculate ganglion.

In a majority of cases of temporal bone fractures, decompression of the labyrinthine segment, geniculate ganglion, greater superficial petrosal nerve, and horizontal facial nerve is sufficient to obtain good facial nerve recovery. In cases of multiple fracture lines or isolated fracture lines which involve other segments of the facial nerve, the nerve can be traced distally to the stylomastoid foramen using the same transcanal exposure (Figure 3). To obtain exposure of the vertical segment, the posterior canal wall is drilled under direct visualization by tracing the facial nerve from distal horizontal segment, downwards to stylomastoid foramen.

**Figure 4:**
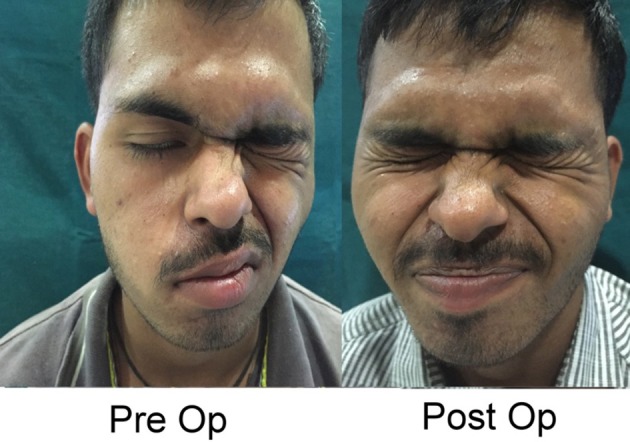
Typical post-decompression, 27 year old man. Pre-operative photograph is 30 days after longitudinal fracture. Post-operative photograph is 6 months after complete transcanal decompression. Permission for publication of these images for educational purposes given by patient and signed by patient and two witnesses, as well at the translator.

After the nerve is exposed and fracture fragments are removed, the epineurium is incised in all cases, using a tenotome. Primary ossicular reconstruction is performed in all cases. Pre- and post-operative air/bone conduction and HB scores (Figure 4) are documented.

This surgical technique requires considerable otologic experience and very accurate anatomic knowledge of the location and course of the labyrinthine facial nerve medial to the geniculate ganglion.

## Results

Table 1 tallies the raw HB scores of 141 patients with post-traumatic unilateral facial nerve weakness grade 4 or worse who underwent decompression from 1998 to 2017. Shown are the segment involved by fracture and their HB scores pre-operatively and 6 months post-operatively. Although bone fragments were removed from several the facial nerve sheathes, none of these 141 patients had a confirmed partial transection. We exclude the one patient from that time period who was found to have a completely transsected nerve.

**Table 1:** One hundred forty-one patients with House-Brackmann (HB) grades HB6, HB5, and HB4 facial weakness after temporal bone fracture—sites of fracture and pre- vs. post- operative HB scores.

**Pt no**	**Site(s) of injury**	**Side Ear**	**Age**	**Pre-operative HB score**	**Post-operative HB score**
1	Perigeniculate	Right	22	5	2
2	Perigeniculate	Left	54	6	5
3	Perigeniculate	Left	53	6	3
4	Perigeniculate	Right	19	4	1
5	Perigeniculate	Left	35	5	1
6	Perigeniculate	Right	38	5	1
7	Perigeniculate	Right	28	6	6
8	Second genu	Right	30	4	1
9	Perigeniculate	Left	50	4	1
10	Horizontal	Right	08	5	2
11	Perigeniculate	Left	27	5	1
12	Perigeniculate	Left	36	4	2
13	Perigeniculate	Right	23	4	1
14	Horizontal	Right	28	5	2
15	Perigeniculate	Left	20	6	1
16	Perigeniculate	Right	26	5	2
17	Perigeniculate	Right	29	5	1
18	Vertical	Left	31	6	2
19	Perigeniculate	Right	29	6	3
20	Perigeniculate	Left	40	6	2
21	Perigeniculate	Right	22	4	2
22	Perigeniculate	Right	17	6	2
23	Perigeniculate	Left	18	5	5
24	Vertical	Right	27	6	2
25	Perigeniculate	Right	50	4	1
26	Perigeniculate	Left	30	5	2
27	Perigeniculate	Right	53	4	1
28	Perigeniculate	Right	29	6	2
29	Perigeniculate	Right	10	5	1
30	Perigeniculate	Left	42	4	1
31	Perigeniculate	Right	40	5	2
32	Horizontal	Left	23	6	5
33	Perigeniculate	Left	24	6	3
34	Perigeniculate	Left	30	5	1
35	Perigeniculate	Right	53	5	1
36	Perigeniculate	Left	24	5	1
37	Perigeniculate	Right	60	6	6
38	Second genu	Right	27	4	1
39	Perigeniculate	Left	30	4	1
40	Perigeniculate	Right	33	5	2
41	Perigeniculate	Right	58	5	1
42	Perigeniculate	Left	52	4	2
43	Perigeniculate	Right	20	4	1
44	Horizontal	Right	30	5	2
45	Perigeniculate	Left	38	5	2
46	Multiple	Right	49	5	2
47	Perigeniculate	Left	42	5	1
48	Perigeniculate	Right	40	6	2
49	Perigeniculate	Left	15	6	3
50	Perigeniculate	Left	47	6	2
51	Perigeniculate	Left	41	4	1
52	Perigeniculate	Right	17	6	2
53	Multiple	Right	23	5	5
54	Vertical	Right	44	6	1
55	Perigeniculate	Left	17	4	1
56	Perigeniculate	Left	18	5	2
57	Perigeniculate	Right	48	4	2
58	Perigeniculate	Right	26	6	1
59	Perigeniculate	Right	24	5	1
60	Perigeniculate	Left	33	4	1
61	Perigeniculate	Right	20	5	1
62	Perigeniculate	Right	30	6	5
63	Perigeniculate	Left	33	5	1
64	Perigeniculate	Right	37	5	2
65	Multiple	Left	33	6	1
66	Perigeniculate	Right	29	5	1
67	Perigeniculate	Right	16	6	6
68	Second genu	Right	30	4	1
69	Perigeniculate	Left	51	4	1
70	Horizontal	Right	64	5	2
71	Perigeniculate	Left	32	5	1
72	Perigeniculate	Right	35	5	2
73	Perigeniculate	Right	24	4	1
74	Perigeniculate	Left	39	4	2
75	Perigeniculate	Left	17	5	2
76	Perigeniculate	Right	28	5	2
77	Perigeniculate	Left	25	5	1
78	Vertical	Right	30	6	3
79	Perigeniculate	Left	44	6	1
80	Perigeniculate	Right	14	6	2
81	Horizontal	Left	20	4	2
82	Perigeniculate	Right	26	6	2
83	Perigeniculate	Left	45	5	5
84	Perigeniculate	Left	31	6	1
85	Horizontal	Left	32	4	1
86	Perigeniculate	Left	29	5	2
87	Perigeniculate	Right	36	4	1
88	Perigeniculate	Right	29	6	2
89	Perigeniculate	Right	48	5	1
90	Multiple	Left	66	4	1
91	Perigeniculate	Right	59	5	2
92	Perigeniculate	Left	42	6	5
93	Perigeniculate	Left	16	6	1
94	Perigeniculate	Right	19	5	2
95	Vertical	Right	22	5	1
96	Perigeniculate	Left	23	5	1
97	Perigeniculate	Right	15	6	6
98	Second genu	Right	48	4	1
99	Perigeniculate	Left	60	6	2
100	Perigeniculate	Right	37	5	2
101	Perigeniculate	Left	45	5	1
102	Perigeniculate	Left	49	4	2
103	Perigeniculate	Right	37	5	2
104	Horizontal	Left	42	5	2
105	Perigeniculate	Left	25	6	2
106	Multiple	Right	45	5	2
107	Perigeniculate	Left	28	5	1
108	Vertical	Right	47	6	2
109	Perigeniculate	Right	38	5	3
110	Perigeniculate	Left	52	6	2
111	Perigeniculate	Left	29	4	2
112	Perigeniculate	Right	33	5	1
113	Perigeniculate	Left	43	5	5
114	Vertical	Right	26	4	1
115	Perigeniculate	Right	24	5	1
116	Perigeniculate	Left	21	5	2
117	Perigeniculate	Left	19	4	1
118	Perigeniculate	Right	20	6	2
119	Perigeniculate	Right	27	4	1
120	Multiple	Left	45	4	1
121	Perigeniculate	Right	34	5	2
122	Perigeniculate	Left	21	6	5
123	Perigeniculate	Left	26	6	3
124	Perigeniculate	Right	31	6	1
125	Perigeniculate	Left	29	5	1
126	Vertical	Right	46	5	2
127	Perigeniculate	Right	31	6	6
128	Second genu	Right	48	4	1
129	Perigeniculate	Left	39	4	1
130	Horizontal	Right	27	5	2
131	Perigeniculate	Left	17	5	1
132	Perigeniculate	Left	23	4	2
133	Perigeniculate	Left	27	4	1
134	Perigeniculate	Right	31	5	1
135	Perigeniculate	Left	29	5	2
136	Perigeniculate	Right	47	5	2
137	Perigeniculate	Left	41	5	1
138	Vertical	Right	38	6	2
139	Perigeniculate	Left	25	5	1
140	Perigeniculate	Left	28	6	2
141	Perigeniculate	Right	32	4	1
	Range			HB6 = 42 patients HB5-62 patients HB4 = 37 patients mean = HB5 (range 4–6)	HB6 = 5 patients HB5 = 9 patients HB3 = 7 patients HB2 = 56 patients HB1 = 64 patients mean = HB2 (range 1–6)

Sites of injury were: the perigeniculate area only (labyrinthine and greater superficial petrosal nerve and geniculate ganglion) = 112/141 (79%); the horizontal segment = 9/141 (6%); the vertical segment = 9/141 (6%); the second genu (5/141 = 4%), and multiple sites—a combination of perigeniculate area with either the second genu or vertical segment = 6/141 (4%). The perigeniculate area, therefore, was involved in 118/141 (84%) of fractures.

On Table 2 the mean (with SD) HB outcomes for patients whose facial function was HB6, HB5 and HB4 on presentation were HB2.79 (1.66), HB1.76 (1), and HB1.24 (0.44), respectively. Fourteen of the 141 (10%) patients showed HB6 or HB5 at 6 months. One hundred twenty of 141 (92%) showed HB2 or HB1 post-operative scores. Just considering the 104 patients with the worst presenting scores of HB6 and HB5: 14/104 (13%) had poor outcomes, and 83/104 (80%) had good outcomes.

**Table 2:** Post-operative HB scores, tabulated by HB score on presentation.

**HB score on presentation**	**# of patients presenting with HB score**	**# of patients with final scores of HB6**	**# of patients with final scores of HB5**	**# of patients with final scores of HB4**	**# of patients with final scores of HB3**	**# of patients with final scores of HB2**	**# of patients with final scores of HB1**
HB6	42	5	5	0	6	18	8
HB5	62	–	4	0	1	29	28
HB4	37	–	–	0	0	9	28
HB3	–						
HB2	–						
HB1	–						
Totals	141	5	9	0	7	56	64

The Analysis of Variance (ANOVA) test demonstrates that these post-operative HB scores differ between the patients whose facial function was HB6, HB5, and HB4 on presentation (f-statistic = 19.05 and *p* < 0.0001). The *post-hoc* Tukey Honestly Significant Difference (Tukey HSD) test, at α-level 0.05 distinguishes between these three presenting patient groups in detail: The outcomes for HB4 and HB5 are each better than the outcome for HB6 (*p* < 0.05), but do not differ from each other.

Table 3 reports average pre- and post-operative air and bone conduction levels parsed by site of injury/decompression. Post-operative bone levels were unchanged. The post-operative air levels were improved in cases of perigeniculate fractures and multiple fractures (perigeniculate plus another site), even after the ossicular disarticulation needed for the approach and the subsequent reconstruction.

**Table 3:** Sites of injury vs. hearing improvement in 140 patients after facial nerve decompression with ossiculoplasty.

**Facial nerve segment involved**	**Number of patients**	**Pre-op vs. post-op air conduction averages (dB)**	**Pre-op vs. post-op bone conduction averages (dB)**
Perigeniculate	112	30–22	14–13
Second genu	5	10–15	5–5
Horizontal	9	19–19	10–11
Vertical segment	9	23–23	12–13
Multiple fractures (geniculate plus)	6	55–25	15–15
Total	141		

## Discussion

Traffic collisions in Bangalore are a major cause of temporal bone fractures with facial nerve palsy. One hundred forty-one cases of traumatic facial nerve palsy with an intact nerve were decompressed at our center through transcanal approach from January 1998 to December 2017. We found our protocol as detailed in the Materials section to very useful both for evaluation and treatment. We do not use electrophysiologic testing, but we have found that the Schirmer's test helps identify injuries proximal to the greater superficial petrosal nerve. And we have found that failure to achieve eye closure at 3 weeks indicates that the patient's course will be better with surgical intervention.

The House-Brackman scale as modified in 1984 is the standard facial nerve instrument approved by the American Academy of Otolaryngology–Head and Neck Surgery, but presents limitations, nonetheless (7). Chief among these are inter-observer differences as well as the ambiguity of scoring secondary effects like synkinsis, contracture and spasm (7). Therefore, in summarizing the results of this series, to be as rigorous as possible, we count HB6 and HB5 outcomes together to find that 14/141 patients (10%) were left with substantial disfigurement after decompression. Considering only those patients with the worst prognoses: 14/104 patients (13%) presenting with HB6 or HB5 had poor outcomes. Similarly, combining HB2 and HB1 as good outcomes, we found that 120/141 patients (92%) had favorable post-operative outcomes. Or that 83/104 (80%) presenting with the worst prognoses—HB6 and HB5—had good outcomes. Statistical analysis found that patients presenting with HB6 had worse outcomes than those presenting with HB5 or HB4.

Comparison to summaries of the literature finds that these results seem slightly more encouraging than previously reported. Adgebite et al. (8) and Brodie and Thompson (9) disagree on the relative prognosis of immediate vs. delayed paralysis after temporal bone trauma. Outcomes as reported in the literature are further complicated by the tendency to combine HB2 and HB1 (9) as we have done here, or to combine HB2-HB5 together (10) as in a large meta-analysis of 35 patient series. With these confounding factors, along with the inter-observer problems with the HB scale, it seems that 0–50% of observation-only post-traumatic paralyzes (9, 10), 44% of steroid-treated paralysis (10), and 21% of decompressed (10) paralysis had HB1 outcomes; while 2, 0, and 10% of these three treatment options had HB6 outcomes (10). In our series 80% of the HB6 and HB5 patients ended with HB2 and HB1, while 13% of these most affected patients ended with HB6 or HB5. Unfortunately, we have no control group to compare the post-decompression results to. However, these results compare favorably to observation-only, the steroid, and the decompression reports in the literature above.

Our patient series shows that the perigeniculate area was involved in 84% of these 141 cases. This area can be reached via the middle cranial fossa. However, the transcanal technique, though technically challenging, gives access to the geniculate ganglion and the labyrinthine facial nerve without craniotomy. In addition, as the improvement in the air conduction scores demonstrates (Table 3), the disarticulation of the ossicles, with their subsequent reconstruction actually improves the post-operative hearing. Most cases have pre-existing disarticulation of the ossicles due to impact of trauma (As an aside, the middle cranial fossa approach affords no access to the ossicles which are often dislocated in temporal bone fracture). Another advantage of this technique is that the routes of the fracture line can be exactly delineated while the corresponding facial nerve pathology can be addressed. Therefore, the transcanal approach avoids a craniotomy, improves the hearing in more than 80% of the cases, and provides exact visualization.

The most frequent objection to this technique is the belief that the labyrinthine portion of the facial nerve cannot be accessed through this approach. Careful practice in the temporal bone laboratory using high magnification will demonstrate otherwise. One has to practice this technique by doing multiple temporal bone dissections before performing live surgeries.

## Data Availability Statement

All datasets generated for this study are included in the manuscript/supplementary files.

## Ethics Statement

The studies involving human participants were reviewed and approved by Vijaya ENT Care Center Ethics Board Committee. Written informed consent for participation was not provided by the participants' legal guardians/next of kin because: This is a review of medical records. As a review of medical records, it was reviewed and approved by the IRB committee. As far as the actual operations—parents and patients gave full informed consent for medical and surgical care. Also the sole facial photograph was obtained with explicit written consent for publication. Written informed consent was obtained from the individual(s) for the publication of any potentially identifiable images or data included in this article.

## Author Contributions

VH assembled this post-traumatic facial nerve protocol from his decades of experience in Bangalore. VV assisted in patient management and together with NM assembled the data and prepared the tables. NM assembled the patient data, prepared the tables, and wrote the first draft of the introduction and materials and methods. MR wrote the final complete article, creating the presentation format commonly used for article submission.

### Conflict of Interest

The authors declare that the research was conducted in the absence of any commercial or financial relationships that could be construed as a potential conflict of interest.
